# Wonderful

**Published:** 1861-09

**Authors:** 


**WONDERFUL.**—According to the latest and most authentic advices on the subject, the greatest, if not the only, wonder that ever appeared in heaven, was a—woman. (See Revelation, 12 : 1.) There was "another wonder," a great red dragon, but it didn't attract particular attention; the "great" wonder was the—"woman." *Per contra*, the greatest mundane wonder we ever met with in the whole course of our life, was a woman, too! This very day, and on this wise: Wifey is out of town, and feeling free-and-easy like, just as we used to in happy days departed, in good old New-Orleans, we thought it no harm to make an appointment to meet a lady in the precincts of "the Avenoo." The clock tolled the hour and the minute as the hand was laid on the bell-pull. The door opened, and there she was on the stair, all ready for the Branch, her husband being out of town. Said she: "My husband was never kept waiting for me a single minute in his life."

In the most serious manner possible, if a man can be serious when there is nobody around to keep things so everlastingly prim and strait-jackety, we repeat, reïterate, and reäffirm the sentiment, idea, fact and faith, that there is not another woman in the whole universal world who can lay her hand on the place where the heart ought to be, and truthfully asseverate, declare and affirm that she never kept her husband waiting for her a minute. We confidently appeal to Barnum, and the rest of mankind, if there is a second to the phenomenon discovered by us at three P.M. this thirtieth day of July, Anno Domini eighteen hundred and sixty-one. Away out West, where we used to navigate round a long time ago, the measure of a man's "how," in the question, "How do you do to-day?" was his being more or less "Bilious!" If "very bilious," he was sure to be as sick as a dog, or as cross as a female bear who had lost her cubs; and he was dangerous. We have seen men as mad as a March hare, and, what was more, obliged to keep mum, and appear very affectionate and polite, while waiting for their wives, who had been "coming in a minute" for the last half-hour.

But about the bile and the legitimate connection of this article with a JOURNAL OF HEALTH and the lady who never kept her husband waiting. By the way, our governor can say the same thing: She is "another wonder," (verse 3,) but the reader will please understand "no connection with" the personage named in the next line. It is meant simply to say she is "another wonder," in that she "never kept her husband waiting a minute." "We don't do things that way in Ningpoo," as the "Japs" used to say, when some of our follies or stupidities attracted their attention. If the very minute comes, and nobody comes with it, Time and I trot on, for it is better for only one to be too late than two. But, as we were saying about the connection between woman, biliousness and health, it is simply this—violent anger has made a man as "yellow as a pumpkin" in an hour: choler causes bile, and bile causes disease. So that if a man wants to avoid an important cause of disease, let him marry a woman who won't "keep him waiting" when they have occasion to go out any where; and we will assure him that the exercise necessary to be taken in order to secure such a phenomenon, will keep him in good health the longest day he lives. Hence, to make the whole thing unmistakably clear, if you don't want to get bilious, don't get mad; if you don't wan't to get mad, get a wife who is always ready, who never keeps her husband waiting a minute. By the way, as this September number may not be out of date before the upper house returns to town, and in order to bring up all things on the square, we desire to say that the lady wonder aforesaid was a grandmother.

